# Cognitive and neural mechanisms underlying false memories: misinformation, distortion or erroneous configuration?

**DOI:** 10.3934/Neuroscience.2023020

**Published:** 2023-09-07

**Authors:** Antonio G. Lentoor

**Affiliations:** Department of Clinical Psychology, School of Medicine, Sefako Makgatho Health Sciences University, South Africa

**Keywords:** true memory, false memory, neural mechanism, mis-remember, misinformation, erroneous recall, hippocampo-neocortical circuits, anterior prefrontal cortex, dorsolateral prefrontal cortex, neuropsychology, cognitive neuroscience

## Abstract

Errors can affect our memory, yet even when there are gaps in our recollection of events, memory often serves us fairly well. Memory formation involves at least three different sub-processes, that are regulated by an underlying neural structure. From a cognitive neuropsychological perspective, a complex process of encoding, consolidating, and retrieval is involved in remembering an event, and it might be hindered by one's emotional state, physiological response to the event itself, and misinformation. As a result, it is very likely that one may struggle to remember specifics of what happened which can increase our susceptibility to the formation of false memories. This has major implications for everyday functioning, as in the case when you mistakenly remember you took your pills when you never did, or where errors have led to false accusations about trauma or abuse, and wrongful convictions of crimes. Memories sometimes contain biases and inaccuracies that prevent them from accurately recalling events. The review will provide an updated overview of current research advances on the cognitive and neural mechanisms underlying inaccurate, distorted, or false memories.

## 1. Introduction

Memory is one of the most essential abilities of the human brain. There is no learning without memory and without memory we are nothing more than unconscious primitive reflexes and stereotypies [1]. Humans rely on memory to perform many cognitive tasks that are basic to human behaviors. However, the process and mechanism of memory is complex and involve activation of multiple brain regions and an interconnected neural network that is not immune to deficits or impairment [2]. Various approaches have been used to understand the mechanisms underlying this process. Recent neuroscience advances including, neuroimaging, electroneurophysiological techniques, and refinement of neuropsychological testing have allowed researchers to reveal new insights into complex and dynamic associations at cellular and structural levels on the neurobiology of memory [3]. As such the pivotal question, as to whether brain activity during the initial memory encoding and consolidation or long-term retrieval differs in relation to whether items are subsequently remembered or missed, is a possibility to be explored [4]. Neuroimaging, in particular functional magnetic resonance imaging (fMRI) [5] and neurophysiological measures such as intracranial electroencephalogram (iEEG) [6], has allowed researchers to extend behavioral research and establish a relationship between generalized patterns of brain activity and cognitive processes that support memory formation and retrieval [7]–[9].

Research in cognitive neuropsychology showed that memory as a reconstructive process can at times be prone to errors or distortions involving a disruption in one or more of the stages (encoding, consolidation, retrieval), and deficits in one of more brain structures, which makes people more susceptible to false memories [10]. False memory typically refers to flaws in remembering [11]. This may be when we remember events in a distorted manner or come to have memories of events that never took place [12]. Erroneous remembering can in part be a result of misinformation or from misremembering.

False memories should not be mistaken for lies nor are they intentionally produced, but it is rather due to error of omission and/or commission [13]. This could entail mixing up details from one event with details from another, or even recalling partial or whole recollections of an incident that has never happened before [14],[15]. Thus, a complex process of encoding, consolidating, and retrieval is involved in remembering an event, and it might be hindered by one's emotional state and physiological response to the event itself and the integrity of the underlying brain structure responsible. Memory as a reconstruction process that is prone to distortion can have severe consequences in our daily lives [16], as in the case of eyewitness testimony, or where errors have led to false accusations about trauma or abuse, all of which can result in wrongful convictions [17]. Examples in day to day lives could include mistakenly remembering taking your pills when you never did, or incorrectly remembering that you locked the car before leaving the parking bay or misremember switching off the stove when you did not do it [18]. False memory appears in our daily lives and it can have significant legal, health and social implications.

False memories have been the subjects of research for a long time. Early investigations have primarily been focused on the behavioral methods of investigations rooted in behavioral psychology. However, during the last two decades, there has been a notable increase in neuroimaging studies concerning the neural underpinning of false memory, allowing at least preliminary links to be made between behavioral and neural processes associated with memory errors. Straube [19] reviewed some of the literature on these studies available at the time. While informative, since then a notable number of fMRI studies investigating the underlying neural basis of false memories have been conducted. Considering the contribution of these neuroimaging studies to our understanding of the neural basis of false memory, a synthesis of these findings is needed. As an attempt to consolidate some of these studies, this paper will provide a narrative synthesis of the cognitive and neural underpinning of erroneous remembering (i.e. false memory).

## 2. Memory as adaptive-reconstructive

Memory represent an information processing system, that we often compare to a computer [20]. However, despite the analogy of human memory to a computer, evidence from neuroscience has shown that a highly complex and interconnected neural network that relies on synaptic activation and modification is involved in memory [21]. The ability to encode, consolidate, and retrieve information from sensory inputs is processed in transient buffers through several key interconnected areas of the brain involving the prefrontal cortex, cortex, hippocampus, cerebellum, and amygdala [22]. This dynamic neural process relies on synaptic plasticity for intercellular transmission and communication [23]–[25].

One of the earliest and perhaps the most classical cases, patient HM in the 1950's, revealed the complexity of the memory system and the importance of the medial temporal lobe for the formation of conscious memory [26]. Decades later, as science methods and neuroscience technology advanced, memory was classified into conscious and unconscious categories, which has become the foundation for understanding the workings of the human memory system. Semantic memories—factual knowledge of the outside world—as well as episodic memories—memory for particular events remembered at a particular time and place—make up conscious memories. However, another type of memory not available to consciousness, often referred to as procedural memory is also important for example using a previously learned skill. Through research, we have come to understand that there must be separate mechanisms for declarative and procedural memory. The empirical data suggest that declarative memory is highly dependent on the integrity of the cerebrum and hippocampus, while the cerebellum is important for procedural memory.

Memory resources are flexible, and adaptable and can be modulated by cognitive factors [27], individual differences, and personality characteristics [28] For example, time, age, attentional capability, emotional valance and arousal, and familiarity with the memory content are some of the many variables that might affect one's ability to recall memories accurately [12],[29]–[32]. Time is particularly interesting and ubiquitous given the ever-changing feature of our experiences and its relevance to the memory paradigm such as learning and forgetting [27],[33]. The available time to encode an experience seems to be important, in that, the length of time spent on encoding an experience does influence the accuracy of memory recall [34]–[36]. Aging has also been linked to increasing susceptibility to memory distortion and inaccuracies with various neural mechanisms implicated (see [37]). This link between aging and erroneous memory can be attributed to several factors such as increased errors in misattribution of memory with aging, familiarity-based errors, imagination inflation, and misinformation. Several studies showed that attentional preference for information must be prioritized in order to be remembered [38],[39]. People prioritize the encoding of information and this is often done so selectively if it has a higher level of importance. On the basis of one's goal and motivation the time spent on selectively rehearsing of information will determine the accuracy in remembering. In addition, compared to non-emotional stimuli, emotional stimuli are preferentially attended to and processed, increasing the likelihood of successful memory retrieval. The evidence suggests that both valence and arousal intensity of emotional stimuli are crucial in the enhancement of memory [12]. Some prior research showed that valence stimuli and emotional events tend to be better remembered than neural information. The research proposed that the explanatory mechanism underlying this is related to the rehearsal and elaborate processing involved in the enhancement of memory for emotional stimuli compared to neutral stimuli [38]. Other evidence refers to the fact that emotional stimuli can be processed automatically. The evidence therefore suggests that negatively-valence stimuli show higher levels of false recall and false recognition than neutral or positively-valences ones. Alternatively, false memory is more strongly influenced by negative positive emotions, or remembering negative events can stimulate higher levels of false memory [39]. A systematic review by Sharma and colleagues [40], showed that anger and greater cortisol response to stress may increase susceptibility to misinformation. Memory enhancement for emotional information appears to happen due to affective processing, the amygdala, and orbitofrontal cortex modulation that facilitate encoding of sensory content and memory consolidation. Notably, the literature is in agreement that while memory is often adaptive and constructive in nature [41], this also makes it prone to mistakes and distortions [42].

## 3. Theoretical perspectives on false memory

It has been proposed that a false memory is a result of flawed memory reconstruction, excessive reliance on familiarity and gist in the absence of accurate retrieval, as well as a flaw in retrieval monitoring (see [43]. As such, researchers have investigated and shown that false memory can be easily induced experimentally in research to participants under various manipulations using variants of the Deese-Roediger-McDermott paradigm. In the literature, the Deese-Roediger-McDermott (DRM) paradigm [3], is one of the earliest and most frequent behavioral psychology methods used to study false memory. Deese-Roediger-McDermott paradigm generates false memories based on semantic similarities across stimuli. Participants are presented with a list of words (e.g., bed, dream, nightmare, night) formulated around a critical lure, a word not appear on the list but that is semantically associated (e.g., word such as sleep). Some of the versions of the DRM paradigm used a recall test instead of a recognition test, with the latest incorporating neuroimagery and electroneurophysiological techniques [44],[45]. Some participant endorses the lure of ‘sleep’ despite not having it in the initial list, a retrieval representing a *false memory*. During the recognition and recall test, the researchers simultaneously look at the neural areas that are active in conjunction with the wrong answers, i.e. false memory effect. Based on the DRM paradigm, false memory can result due to word association strength and gist. Evidence from these studies provided an understanding of key areas in the brain that appear to be activated during misremembering.

The two popular theories of the DRM that explain how and why the brain organizes information in a way that might occasionally cause memory mistakes are the Fuzzy-trace theory and the Activation-monitoring theory (see [42]. The Fuzzy-trace theory (FTT) explains the formation of false memory based on gist (the meaning of an event) or fuzzy trace connections that fuel false memory in the absence of verbatim information [43]. Based on FTT we encode and store in parallel two distinct traces of an item, the verbatim trace representing the surface form of the word, whereas the gist trace preserves the semantic features, such as the meaning of the event, for example, gist remembered *sleep*—to take rest, mainly at night. False recollections are based on meaning (gist), especially in the absence of verbatim information [29]. This is often referred to as a dual process approach because recollection of verbatim content suppresses false memories.

On the other hand, as per the Activation-monitoring theory (AMT), the terms “activation” (primarily happens at encoding) and “monitoring” (largely at retrieval) specifically refers to the decision-making processes that aim to identify the source of the activated concept and a stimulus that promotes the associative activation between words [46]. This theory proposed that false memory occurs when participants fail to monitor the source of the activated item, and thus mistakenly remember the critical lure that was generated externally (from the list) rather than internally (from spreading activation). Any disruption in the source monitoring has been shown to increase false memory. These theories, which suggest that memory errors may be connected to the encoding, consolidation, and/or retrieval processes, have received substantial support over the past 20 years from numerous studies that look at the underlying brain activity implicated in false memory (see [47]).

## 4. Encoding and underlying neural process

The brain takes in sensory information from the world, it organizes it stores information in order to be remembered in later memory systems, a process known as encoding. This often involves multisensory information that is automatically and transiently processed, and collated with previous semantic knowledge to form episodic memories during encoding [48] The brain is able to categorize information later on using attentional processes to further prioritize it into short-term memory stores [49]. The act of pulling the knowledge out of memory and back into awareness which was encoded and stored is referred to as retrieval [27]. These different systems involved in the memory formation process have been shown to be vulnerable to errors, and distortions that can result in false memories [18]. Several studies implicate that declarative memory which is a long-term human memory system that involves recognition, encoding, and retrieval of conscious knowledge, facts, or events is particularly vulnerable to errors and distortion which has been associated with false memory formation [50],[51]. More specifically, episodic memory compared to semantic memory (i.e. facts about the world) system has been shown to be more vulnerable to distortions [52]. These may have major implications for an individual.

In the legal context, eyewitness plays an important role in testimony that can either lead to convictions or freedom for an individual. Despite the persuasive role of eyewitnesses their memories sometimes can contain biases and inaccuracies that prevent them from accurately recalling events. Eyewitnesses tend to remember incomplete or false events, as well as particular facts incorrectly. At least in part, mechanisms that occur during encoding are to blame for erroneous recollections. The research showed that prior knowledge, mental state, emotions, and context influence memory and thus what is retrieved from memory can be significantly different from what initially was encoded [50],[53],[54]. Thus, false memories are widely acknowledged to be a significant unintentional result of elaborate semantic and visual encoding processes.

Neuroimaging evidence suggests that processes like imagery, self-referential coding, or spreading activation may lead to false memory formation during encoding [55]. The involvement of different neural structures [10], including occipital and parietal structures, is particularly relevant in the formation bias in memory. Some evidence suggests that similarities between imagined and perceived events encoded may cause errors that result in confusion at retrieval and may induce memory bias or false memories types, this is particularly associated with activation predominately in the occipital and inferior parietal regions, such as the precuneus [48]. Functional magnetic resonance imaging (fMRI) studies involving the processing of self-referential stimuli showed that the cortical midline structures (CMS), specifically the anterior cingulate cortex (aCC), middle temporal gyrus (mTG), anterior/dorsolateral prefrontal cortex (a/dlPFC) and ventromedial prefrontal cortex (vmPFC) are involved in formation erroneous memory or false memories at encoding [56],[57]. The a/dlPFC), vmPFC and more specifically, the hippocampus are believed to be responsible for associative memory, specifically binding memory. Particularly, the hippocampus appears to be critically involved in the misinformation effect. As such, associative decline linked to reduced a/dlPFC and vlPFC activation has been shown to enhance susceptibility to false memories. Current evidence suggests that in part the vulnerability to the formation of false memory is linked to binding failures during encoding which can lead to the loss of distinctive memory sources that contribute to misattribution between different memories due to misinformation. These neural structures are thought to work in concert during to formation of false memories [10],[58].

## 5. Consolidation and underlying neural process

Existing research showed that new memories must be interleaved within a large network of relevant pre-existing knowledge. New consolidated memory therefore could be erased during memory re-organisation to incorporate new learning. Integrating new with old information is a process that involves an ever-evolving organization of experience. After learning, information is susceptible to interference for some time. As memories consolidate (stored), they may become distorted as a result of new information, sleep or lack thereof, or both. For those distortions, various mechanisms have been hypothesized to be relevant. For example, sleep has been shown to play an important role in the consolidation of declarative memory. However, sleep may influence the creation of false memories [29] through semantic generalization through active reorganization of memory traces in the post-learning sleep period. New incoming information may be prone to distortion during consolidation due to retroactive interference (i.e. when memory is changed by new incoming information at consolidation). This might lead to the retrieval of incorrect information that was introduced after the occurrence as if it had truly occurred, which is known as the misinformation effect [59],[60]. Newly acquired information is highly labile and susceptible to the influence of external factors that may enhance or impair long-term retention.

Sleep plays an important role in memory due to its influence on how the brain functions. Disturbances in memory consolation as well as retrieval have been shown to be strongly associated with sleep deprivation and the same mechanism may be responsible for false memory formation [19]. Sleep deprivation seems to be associated with misinformation because of memory trace impairment. Notwithstanding, some evidence showed that sleep, relative to lack of sleep, enhances accurate and false recollections [61],[62]. Moreover, when misleading information is presented before consolidation, sleep may integrate misleading information into memory for real events, increasing distortion. It is well recognized that sleep creates the ideal neurobiological environment for consolidating memories for long-term storage, but sleep deprivation can severely hinder memory retrieval. Furthermore, loss of sleep or sleep deprivation reduces the amount of information we can store in our working memory, may impair one ability to learn, and negatively affects cognition. A study by [63] found sleep deprivation increased false memories in a misinformation task when participants were sleep-deprived during event encoding, but did not have a significant effect when the deprivation occurred after event encoding. Neuroimaging evidence showed that sleep seems to boost systems level consolidation of memories in the hippocampo-neocortical circuits that are ultimately linked to both correct and false memories rather than specifically enhancing false memories [34],[64],[65].

## 6. Retrieval and underlying neural process

Not only are memories vulnerable to incorrect encoding and forgetting, but memory retrieval is also very susceptible to errors [66],[67]. Reconstruction determines how accurately we remember. Therefore, understanding how false memories occur requires knowing the reconstruction process in retrieval [3]. Due to its reconstructive nature, memory is susceptible to misattribution, which occurs when a specific memory is binding to the wrong moment, location, or subject. Reconstruction of memories during retrieval is based on the source monitoring framework [44], which allows for the introduction of numerous defects, such as aspects of comparable events or imagined events that interact to create an erroneous recollection. When events are remembered with great sensory detail and falsely believed to have actually happened, inaccurate memory may be the result of a failure in reality monitoring.

Previous research has connected a failure in reality monitoring to inaccurate memory, which happens when experiences are remembered with great sensory detail but incorrectly believed to have truly occurred [12]. This is partially explained by a brain function that involves mental imagery and may be related to perception. Therefore, it is conceivable that the overlap may lead to imagined occurrences being mistakenly taken for real, and this susceptibility to mistaken attribution could be heightened by suggestive and false information [17]. False memories can result from incorrectly attributing incorrect information to the original event, which lowers the possibility that true memory retrieval will occur [52]. The evidence showed that even the subtlest forms of incorrect or misleading information can significantly alter memory for past events [68]. Memory distortions due to misinformation have been linked to faulty reconstructive processes during memory retrieval and the reactivations of brain regions involved in the initial encoding of misleading details (cortical reinstatement). This does not appear to differ for a subgroup of individuals with Highly Superior Autobiographical Memory (HSAM), who have recently been revealed in the scientific literature to possess a remarkable and exceptional ability to recall details with astounding accuracy [69]. Notably, despite prior research [60] showing enhanced behavioral and neurobiological mechanisms for consolidating and retrieving episodic autobiographical information [70]–[72], people with HSAM are still likely to be susceptible to fallible reconstruction processes when presented with misinformation. They are therefore as likely the typical person to remember false memories [60]. In line with theory, misinformation errors may be due to the faulty reconstructive nature of memory retrieval, in that misleading information is retrieved from memory and misattributed to original source information. This failure in source monitoring results in inaccurate details about an event being reactivated during retrieval [47].

A number of studies over the last two decades have explored the misinformation effect (see review [59]. Misinformation comes in many forms and it has significant clinical and legal implications. Everyday life could be influenced by misinformation. According to the existing evidence age [73] and personality [28] are some of the mediating factors for a person's susceptibility to misinformation effect. Understanding how the brain changes with age and how these changes affect memory, recollection, and false memories has garnered a lot of attention recently. This body of research suggests that associative binding and strategic monitoring are significantly affected by age-related decreases in the medial temporal and prefrontal brain regions, as well as other age-related changes [37]. Misinformation effect is a phenomenon that commonly occurs in eyewitness testimonies, for example, memory errors caused by incorrect information between the original event, such as crime, and later memory, such as an interview lineup or day in court. This can lead to inaccurate memories and in some situations result in the formation of false memories. This is particularly true after exposure to misleading or erroneous information. For example, researchers have shown that questions asked after witnessing an event can actually influence a person's memory of that event, especially of the question contains misleading information which can distort the memory of the event or even remember an entire event that did not actually happened (see [59],[74],[75]).

A recent meta-analysis furthermore showed that fMRI is useful for discriminating between deception and false memory, which is important given the reliance on eyewitness testimony in legal proceedings [5]. In their research on the misinformation effect, cognitive psychologist Elizabeth Loftus and colleagues demonstrated that misleading information can actually overwrite the original memory of an event. People are more susceptible to distortions in their recollection after exposure to misleading information [56] and with increasing age, this vulnerability is heightened. Some of the evidence showed that errors in encoding of original events may be misplaced by misleading information to fill in gaps in memory and thus make an individual susceptible to recalling false memories. In line with previous studies, Zhuang and colleagues recently showed that during retrieval new information could be updated from the current context which provides additional cues to recall target memories. However, the integration of new information during retrieval might generate mismatched contextual memories, thereby producing false memories in recall.

The current research showed that neural processes associated with different prefrontal cortical regions are differently involved with memory retrieval, according to previous neuroimaging research using positron emission tomography and functional magnetic resonance imaging. The research suggests that, in addition to its long-standing involvement in accurate recall, the medial temporal lobe (MTL), and specifically the hippocampus appears to be a critical factor in misinformation formation and recall [10],[18]. Studies have shown that the MTL is important for memory reconstruction and for discriminating true memory from false memory [76]. Other research revealed that during false recognition, brain activity increased in the medial and lateral frontal cortex [3]. In the posterior sections of the parahippocampal gyrus and the occipital visual regions, activity for real memories was greater than for false memories, according to a study by [56] while the left precentral gyrus, medial superior frontal gyrus, and left inferior parietal cortex were more active for false memories retrievals than for true memories [47].

## 7. Conclusions

Collectively, the evidence showed that memory is a malleable reconstructive process with dynamic and intricate underlying neural mechanisms. The review showed that memory formation involves at least three sub-neurocognitive processes, each of which is susceptible to particular biases, distortions, or errors that could lead to erroneous recollections. Notably, neural correlate studies to date support theories that explain false memories, including the fuzzy trace theory, which considers gist-like signals, and the spreading activation, which holds that false memories are caused by a difference in the strength of signals between encoding and retrieval. The most consistent conclusion based on existing fMRI studies suggests that false memory may be related to the lateral prefrontal, medial prefrontal, and medial temporal lobes, including the hippocampus and parahippocampal gyrus.

The real-life implication of distortions or erroneous memories is that they can affect our present-day perception and how we react to the past as well as our judgments and preferences for the future. It can lead to errors and bias that can result in wrongful convictions, in the case when having to give eyewitness accounts to crimes, with negative life implications affecting everyday functioning.

Recent brain imaging and neurophysiological studies have attempted to provide novel ways of investigating memory which has allowed for new insight into the underlying neural mechanisms and cognitive processes of false memory formation and retrieval, and the correlating neuroanatomical substrates. By building on earlier studies on false memory [19], these researchers [3],[77],[78] and other cognitive neuroscientists have been able to use state-of-the-art imaging techniques to understand false memory development. These findings, if anything, add to behavioral research and advance use close to figuring out whether there are brain circuits that may be able to recognize false memories and how this process works. Due to advancements in neuroimaging, this topic is now being studied more in-depth. Future studies could include more cost-effective and feasible brain imagery techniques such as functional near-infrared spectroscopy (fNIRS) during DRM paradigm tasks. Even though these findings mark a significant advancement in our understanding of the neural processes underlying false memory, further research is necessary before we can draw any clear conclusions.
